# Presence of Orthopedic Residency Decreases Emergency Physician’s Confidence in Orthopedic Procedures

**DOI:** 10.7759/cureus.15551

**Published:** 2021-06-09

**Authors:** Eric Blazar, Bernard Jones, Babak Behgam

**Affiliations:** 1 Emergency Medicine, Inspira Medical Center Vineland, Vineland, USA; 2 Emergency Medicine, OhioHealth Doctors Hospital, Columbus, USA

**Keywords:** residency, emergency medicine resident

## Abstract

Introduction: At present, there exists no standard orthopedic training for emergency medicine (EM) residency programs. Varying residency environments including but not limited to volume, acuity, and competing residency programs will dictate the number of orthopedic procedures a resident is exposed to, ultimately dictating a graduate’s comfort level with orthopedic procedures. Our study set out to investigate further whether training alongside an orthopedic residency affects an attending physician’s perceived procedural comfort.

Methods: This is a cohort study utilizing a 14-question survey distributed to an online community of EM physicians to examine the relationship between training at a residence program alongside orthopedic residents, the utility of an elective orthopedic rotation, and the overall confidence in managing closed reductions.

Results: EM physicians trained at a program that also hosted an orthopedic residency were more likely to train at large academic tertiary care centers (78%). Forty-two percent of these respondents felt that the presence of an orthopedic residency had a negative impact on their overall orthopedic training. The remaining 58% felt that the orthopedic residency had a positive impact on their procedural skills. In our study, the overall mean comfort level was statistically significant (p-value = 0.0024) higher in those who trained without orthopedic residents (8.78) compared to those who trained alongside an orthopedic residency (7.61). Those who had an elective orthopedic rotation found it to be more beneficial if they did the rotation with an orthopedic residency (p-value = 0.0329). Those who reported a beneficial orthopedic rotation also reported a higher level of confidence in the management of non-fracture reductions (p < 0.011, ρ = .25).

Conclusion: Seeing as though both training and practice environments for emergency physicians vary greatly across the country, every effort should be taken to ensure graduating residents are prepared to perform orthopedic procedures without the assistance of orthopedic surgeons. Irrespective of whether a program has in-house orthopedic residents or not, EM residents should take it upon themselves to maximize their time during residency to focus on these core competencies. Graduates at the greatest risk of having low confidence are trained at academic centers that also host orthopedic programs. One should be cognizant of this while going through their EM residency.

## Introduction

Orthopedic injuries account for a large number of annual emergency department (ED) visits. In the United States, 13.9% of ED visits are for musculoskeletal complaints, and fractures alone account for nearly 3% of all ED visits. A 2012 study sought to understand how ED physicians perceived their orthopedic on-call coverage. In that study, only 29% of the respondents felt their orthopedist always came in when asked to evaluate the patient [1]. Furthermore, the American Orthopedic Association has identified diminishing orthopedic surgery coverage among community EDs as a critical issue [2]. This highlights the important role that the emergency medicine (EM) physician plays in identifying and managing acute orthopedic injuries.

Another recent 2016 study evaluated 287 recent EM residency graduates from seven different residency programs to assess their level of preparedness in managing closed fractures upon completion of their residency. The study found that many graduates felt either not at all prepared (12%) or only somewhat prepared (44%) [3].

The level of post-graduate preparedness and comfort level in managing orthopedic and musculoskeletal injuries is multifactorial but invariably stems from exposure to and familiarity during residency training. Orthopedic training during EM residency is highly variable and is often contingent upon each individual residency program. Most programs utilize a mixture of a formal lecture, self-study, simulation, and on-shift experience.

Numerous studies have highlighted the apparent lack of proficiency in orthopedics among EM physicians. These studies highlighted the need to revise the orthopedic curriculum for EM residents through increased exposure to musculoskeletal and orthopedic issues [4]. A previous study published in the Annals of Emergency Medicine asked residents to rate their confidence in orthopedic case management using a five-point scale, from “not confident” to “confident.” Responses were dichotomized (1 to 3, not confident; 4-5, confident) [5]. Another study asked attending physicians to rate their subjective preparation of independent fracture reduction, which gave respondents one of four categories to choose from. These ranged from “not at all prepared,” “somewhat prepared,” “fairly well prepared,” to “very well prepared” [3], with the majority answering that they felt “somewhat prepared” [3].

Many EM graduates train at large academic institutions where orthopedic residents are readily available for ED consultation. Since the studies on EM orthopedic proficiency are thought to be related to inadequate exposure, it stands to reason that training alongside an orthopedic residency may have an impact on post-graduate procedure confidence.

The purpose of our study is to determine what impact, if any, does training in conjunction with orthopedic residents has on post-graduate comfort levels for managing orthopedic injuries. We will also attempt to evaluate the proportion of graduates who had a formal orthopedic rotation as a part of their training to determine whether this had any impact on post-graduate orthopedic procedure comfort.

## Materials and methods

Study design

This is a cohort study in which a survey was completed by currently practicing EM attending physicians who are members of a Facebook group called EM Docs, which is where the survey was distributed (Table 1).

**Table 1:** Survey distributed to EM attending physicians EM, Emergency Medicine; ACGME/AOA, Accreditation Council for Graduate Medical Education/American Osteopathic Association.

Questions	Answers
1. Inspira Medical Center Vineland physicians are conducting an IRB-approved research project to study procedural confidence in the management of orthopedic injuries and the effect that training in congruence with an orthopedic residency has on emergency physician confidence after training. Completion of the study’s survey is voluntary and anonymous. Your name and no identifying data will be included in the report of this study. The act of completing the survey will constitute your consent to participate in this project. You have the option to stop completing this survey at any time. Special vulnerable populations, including those under the age of 18 years of age, pregnant women, prisoners, individuals with mental disabilities or cognitive impairments, individuals with physical disabilities, and individuals who are institutionalized (for example, persons in correctional facilities, nursing homes, or mental health facilities), should not complete this survey. This research has received no fiscal or commercial support of any kind. Would you like to continue?	Yes
	No
2. Did you graduate from a US-accredited ACGME/AOA program?	Yes
	No
3. Did you train in a three- or four-year program?	Three-year program
	Four-year program
4. How many years have you been practicing EM as an attending physician?	
5. How would you describe the primary hospital at which you trained during residency?	Large academic tertiary care center
	Non-tertiary care center with basic residency programs limited to no fellowship or sub-specialty training
	A community hospital with few residency programs
6. Where was your residency located?	Northeast (PA, NJ, NY, CT, MA, VT, RI, NH, ME)
	Midwest (ND, SD, KS, MN, NE, IA, MO, WI, MI, IL, IN, OH)
	South (DE, MD, DC, VA, WV, KY, NC, SC, TN, TX, GA, AL, MS, FL)
	Pacific (AK, HI)
7. Was there an accredited orthopedic residency program at the program at which you trained?	Yes
	No
8. If answered "yes" to number 7, how would you rate the influence that the orthopedic residency program had on your overall orthopedic training?	Negative (It hindered your education and procedural skills.)
	Positive (You learned by training alongside orthopedic residents.)
9. Did you have a dedicated orthopedic rotation as part of your EM residency training?	Yes
	No
10. If answered “yes” to question #9, did you find the rotation helpful? (Please rate on a numerical scale with 0 = not at all helpful and 10 = very helpful.)	
11. What proportion of the time would you say that you need to call your on-call orthopedist to assist you in a non-fractured joint reduction for a native/non-prosthetic joint due to failure to reduce independently? (Please do so by entering the percentage of the time.)	
12. What kind of setting do you currently practice in?	Large academic tertiary care center
	Non-tertiary care center with basic residency programs limited to no fellowship or sub-specialty training
	A community hospital with few residency programs
13. What aspect of orthopedic management causes the most difficulty on your current shifts?	Diagnostic interpretation of x-rays
	Orthopedic joint reduction
	Joint arthrocentesis
	Other (Please describe in question 14.)
14. If you answered "Other" for question 13, please answer in the following text box. What aspect of orthopedic management causes the most difficulty on your current shifts?	

Study population

The respondents of the survey include practicing EM physicians who are members of the Facebook group EM Docs, which currently has over 21,000 members. The survey was open to attending physicians only, and a total of 146 currently practicing EM physicians anonymously completed the survey.

Outcomes measured

The primary outcome of this study is to assess the perceived effect that training at an institution with an accredited orthopedic residency has on the EM physician’s confidence in managing orthopedic injuries.

The secondary outcome of this study investigates the proportion of EM physicians who had a formal, dedicated orthopedic rotation as part of their training and whether this had any effect on their confidence in managing orthopedic injuries.

Data collection

A 14-question survey (Table 1) was distributed among EM attending physicians. The survey asked the responded questions that pertained to where the physicians did their training, their practice environment (community vs. academic), whether they trained in the presence of an orthopedic residency, and whether training in congruence with an orthopedic residency helped or hurt their overall confidence in the management of non-fracture reductions. The survey also evaluated whether, as part of their training, attending physicians had a dedicated orthopedic rotation and whether the rotation was helpful. The results of the survey were recorded in SurveyMonkey (SVMK Inc., California, United States).

Statistical analysis

Summary statistics were provided for continuous variables (n, mean, SD, min, median, max, confidence interval) and categorical variables (frequency and percent). A t-test was used to find significant differences in the means, and chi-square was used to find the association between categorical variables. Pearson’s correlation coefficient was used to examine the relationship between the utility of an elective orthopedic rotation, the overall confidence of the attending physician in managing closed reductions, years practicing, and the proportion of the time that the attending physician had to call in an orthopedist to assist in a non-fractured joint reduction for a native/non-prosthetic joint due to failure to reduce independently.

## Results

A total of 146 EM physicians completed the survey. Among the respondents, 63% completed their residency training in a three-year accredited program, while 37% completed their training in a four-year program. Nearly two-thirds of the respondents trained at large academic tertiary care centers, while the remaining respondents were split evenly between non-tertiary care centers with basic residency programs and community hospitals with only a few residency programs. Residency programs in nearly all geographical parts of the country were well represented. Respondents trained in all parts of the country except for Hawaii and Alaska. The mean number of years of practice among the respondents was 6.6, with a range between 0 and 27 years of practice.

There was a significant association between respondents who trained in the presence of an accredited orthopedic residency during their EM training and the primary hospital in which they trained (p < 0.0001). The survey found that physicians who did not train in the presence of an orthopedic residency were more likely to train at community hospitals (68%), whereas EM physicians who trained in a system that also hosted an orthopedic residency were more likely to train at large academic tertiary care centers.

**Figure 1:**
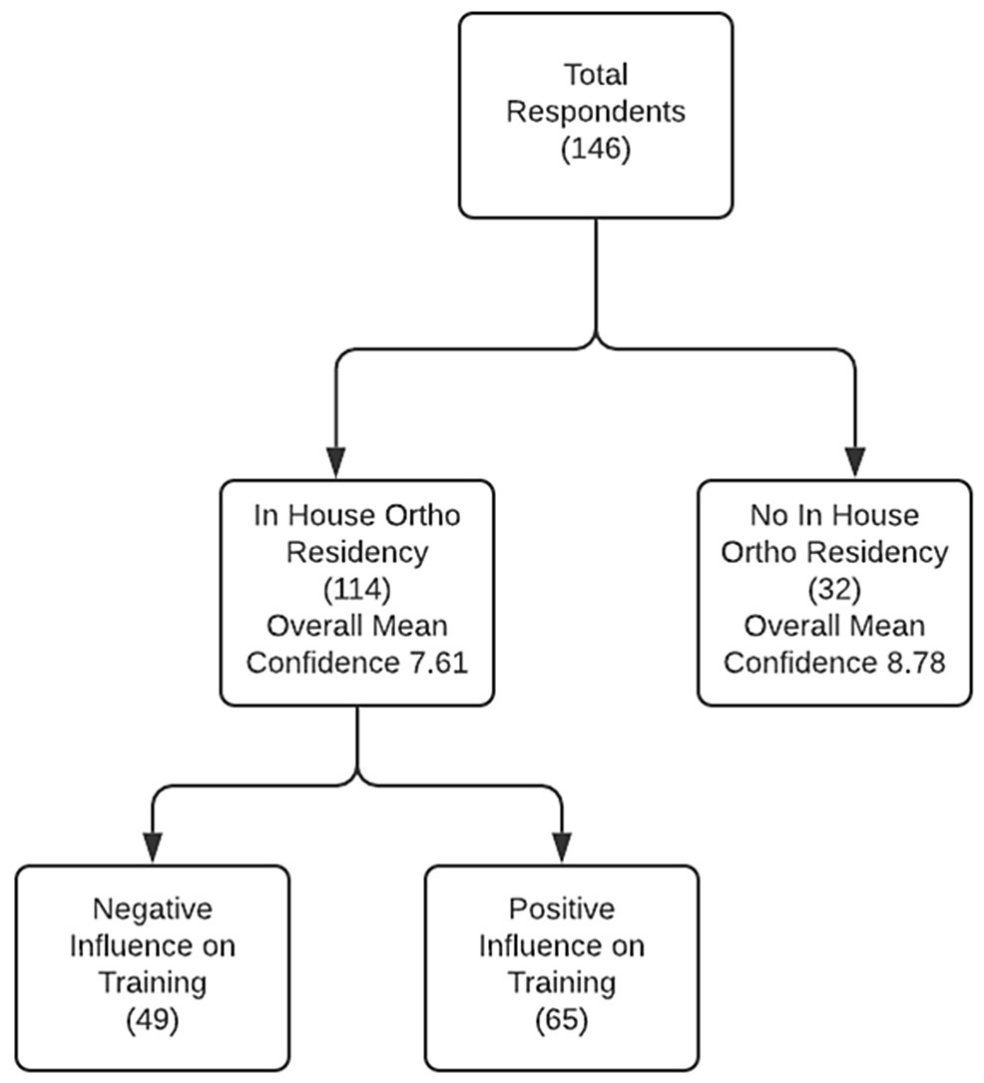
Net effect on confidence due to in-house orthopedic residency program

Among the 146 respondents, 114/146 (78%) trained at an EM residency program that was affiliated with an orthopedic residency. Of the 114 respondents who trained alongside orthopedic residents, 48/113 (42%) felt that the presence of an orthopedic residency had a negative impact on their overall orthopedic training and hindered their educational and procedural skills. One individual who did train in the presence of an orthopedic residency did not respond to the impact that the orthopedic residency had on their orthopedic training during residency. Furthermore, 65/113 (58%) of those who trained in the presence of orthopedic residency felt that the program had a positive impact on their orthopedic procedural skills and they benefited by training alongside orthopedic residents (Figure 1).

Respondents were asked about how they would rate their overall confidence in the management of orthopedic non-fracture reductions without assistance from an orthopedic surgeon. Confidence was measured on a 10-point scale, with zero equating to no confidence at all and 10 being very confident. When comparing the 114 respondents who trained in congruence with an orthopedic residency to the 32 that did not, those who did not train with orthopedic residents had a higher overall mean confidence (8.78) when compared to their counterparts who did train alongside orthopedic residents (7.61). This was found to be statistically significant (p-value = 0.0024) (Figure 1).

**Figure 2:**
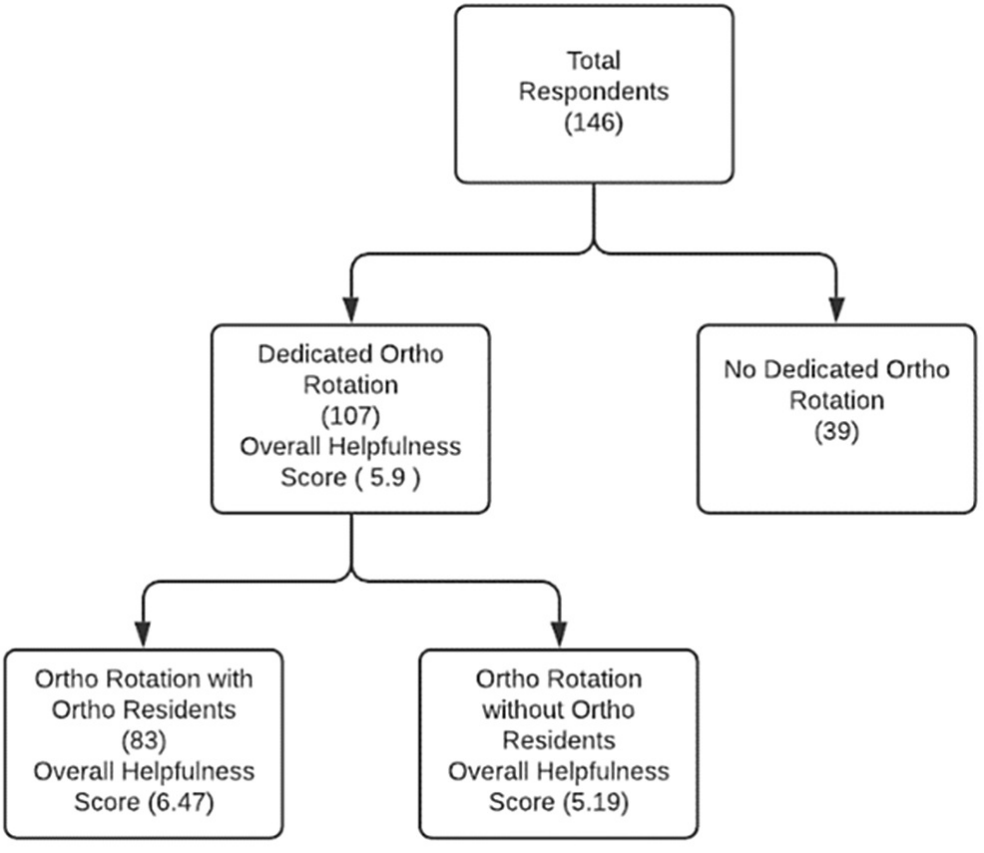
Overall helpfulness score of dedicated ortho rotation

Furthermore, of the 146 respondents, 107/146 (73.3%) reported that they had a dedicated orthopedic rotation as a part of their EM residency training, while 39/146 (26.7%) did not have a dedicated orthopedic rotation. Those who had a dedicated orthopedic rotation were asked to express the degree to which they found the rotation to be helpful, on a zero to 10-point scale (0 = not at all helpful, 3 = somewhat helpful, 6 = fairly helpful, 10 = very helpful). The average response to the degree of helpfulness was 5.9. One person who did have a dedicated orthopedic rotation did not respond to this question (Figure 2).

Among the 106 respondents who did have a dedicated orthopedic rotation, 83/106 (78%) also trained alongside an accredited orthopedic residency, whose mean score, in terms of the helpfulness of the orthopedic rotation, was 6.47. Conversely, 23/106 (22%) of those who had a dedicated orthopedic rotation did not train alongside an accredited orthopedic residency, and their mean response to the helpfulness of a dedicated orthopedic rotation was 5.19. This was less than their counterparts who had a dedicated orthopedic rotation and trained alongside an orthopedic residency, which was found to be statistically significant (p-value = 0.0329) (Figure 2).

There was a strong correlation between how helpful a dedicated orthopedic rotation was and overall confidence in the management of non-fracture reductions (p < 0.011, ρ = .25). The more the respondents felt the rotation was helpful, the higher confidence they had in their abilities.

Lastly, in regard to aspects of orthopedic management that the respondents found to be most difficult, 16/140 (11%) found it to be a diagnostic interpretation of x-rays, 41/140 (29%) found it to be an orthopedic joint reduction, and 36/140 (26%) found it to be joint arthrocentesis. The remaining 47/140 (34%) answered “other” and were given the opportunity to type out their response. Of those 47 respondents who answered “other,” the majority of them, 13/47 (27%) felt that complex fracture reductions were the most difficult aspect of orthopedic case management.

## Discussion

The ability to manage orthopedic injuries is paramount to practicing EM physicians, given the prevalence of these injuries and the diminishing orthopedic coverage in the ED. Studies have highlighted the apparent lack of proficiency in orthopedic case management among EM physicians. The lack of musculoskeletal education in medical school and the impact of residency training are thought to play a role in the ability of the practicing EM physician in managing these orthopedic injuries. This study aimed to identify the impact that training alongside orthopedic residents who are part of an accredited orthopedic residency had on EM physicians' comfort levels as it relates to the reduction of non-fracture dislocations. Among those who trained alongside an orthopedic residency and among orthopedic residents, a small majority (58%) felt that training with orthopedic residents had a positive impact on their orthopedic skills. This did not, however, translate into procedural confidence as an attending, which was measured as a confidence level on a 10-point scale in the management of non-fracture reductions when orthopedics was not available. The study found that those who did not train alongside orthopedics during their residency training had a higher overall mean confidence (8.78) when compared to their counterparts who did train alongside orthopedics (7.61), which was found to be statistically significant.

In terms of the utility of a dedicated orthopedic rotation, the majority of the respondents who had a dedicated orthopedic rotation found the rotation to be somewhat helpful in their orthopedic training. When examined on the basis of having a dedicated orthopedic rotation while also training in the presence of an orthopedic residency, the study found that those who trained alongside orthopedic residency found the rotation to be more helpful than their counterparts who had a dedicated orthopedic rotation but did not train with orthopedic residents.

Limitations

We are cognizant of many limitations in our study. First, the online survey design of the study limited access to the number of EM physicians that we were able to gather for the study. The study was distributed among a group of over 21,000 members who are EM physicians, and 146 physicians responded to our questions. While this group is a decent representation of the EM physician community, a larger sample of practicing EM physicians would have been more helpful to increase the heterogeneity and diversity of the study. Though the group polled is highly policed by group administrators to ensure all members are EM physicians, there is no way to verify who responded to the survey. Second, the conducted study was designed as a survey study. We acknowledge the limitations of this method of study; however, we felt this was an acceptable option to obtain the sentiments of the wider practicing EM physician population with respect to orthopedic comfort and confidence.

Furthermore, the definition of orthopedic procedural confidence, a subjective measure, was limited to how attending physicians viewed their ability to manage non-fracture dislocations. In our study, we used the subjective measure of "confidence" as a surrogate for comfort level. In reality, confidence can pertain to much more than just the ability to reduce non-fractured dislocations. The way confidence is defined could be expanded to include other orthopedic-related procedures. We also note that confidence and comfort are not the same, and having confidence does not mean a physician is comfortable and vice versa. Nonetheless, we felt it was important to report our findings to further help shape the landscape of EM training.

## Conclusions

We found that the majority of responding EM physicians were trained at three-year residency programs at academic tertiary care centers with dedicated orthopedic residents. A small majority felt that training alongside orthopedics contributed to their procedural comfort level as an attending physician. We found that confidence is higher in those EM physicians who did not train alongside orthopedics. This is likely related to the unopposed nature of managing all orthopedic reductions independently during training, in the absence of a dedicated orthopedic resident. Based on our data, it stands to reason that with more independence due to the lack of a dedicated orthopedic residency, EM physicians increase their overall confidence. Having a dedicated orthopedic rotation as part of residency training does seem somewhat helpful to trainees and plays a role in procedural confidence.

Efforts should be taken to ensure graduating residents are prepared to perform orthopedic reductions and manipulations without the assistance of orthopedic surgeons. Whether a program has in-house orthopedic residents or not, EM residents should take it upon themselves to maximize their time during residency to focus on these core competencies. Graduates who are at the greatest risk of having low confidence are those who are trained at academic centers that also host orthopedic programs. Residency leadership and residents should be cognizant of this potential while going through their EM residency. Steps should be taken through both simulation and standardization of education to ensure all graduating EM residents have the ability to practice confidently upon graduation.
